# Large Language Models as Decision Support in Breast Cancer Management: A Comparison with Multidisciplinary Team Decision-Making

**DOI:** 10.3390/jcm15197533

**Published:** 2026-09-28

**Authors:** George Călin Dindelegan, Noé Yoshi François Poupel, George Ionuț Golea, Radu Alexandru Ilieș, Matei George Cristea, Simona Ioana Filip, Gerald Gheorghe Filip, Eugeniu Darii, Iyad Shahin, Octav Ginghină, Alexandra Caziuc

**Affiliations:** 1 Department of General Surgery, “Iuliu Hațieganu” University of Medicine and Pharmacy, 400012 Cluj-Napoca, Romania; 2 First Surgical Unit, Cluj County Emergency Clinical Hospital, 400006 Cluj-Napoca, Romania; 3 Faculty of Medicine, “Iuliu Hațieganu” University of Medicine and Pharmacy, 400012 Cluj-Napoca, Romania; 4 Department of Surgery, Regina Maria Ponderas Academic Hospital, 014142 Bucharest, Romania; 5 Regina Maria Hospital, 500091 Brașov, Romania; 6 Ovidius Clinical Hospital, 905900 Constanța, Romania; 7 3rd Surgical Oncology Departament, Institute of Oncology “Al Testioreanu”, 022328 Bucharest, Romania

**Keywords:** breast cancer, large language models, GPT-5.5, artificial intelligence, clinical decision support, multidisciplinary team, surgical oncology, treatment concordance

## Abstract

**Background/Objectives:** Breast cancer management relies on multidisciplinary team (MDT) decisions that integrate clinical, radiological, pathological, and patient-related factors. Large language models (LLMs) may support such decisions, but evidence based on real-world cases remains limited. **Methods:** We evaluated the agreement between ChatGPT-5.5 (OpenAI, San Francisco, CA, USA) and real MDT management of patients with invasive breast cancer. In this single-center, retrospective, proof-of-concept study, standardized preoperative clinical vignettes for 86 patients with invasive breast carcinoma were independently evaluated by GPT-5.5 (frozen prompt, Medium reasoning mode, Web Search enabled). AI-generated recommendations were compared with the MDT reference standard across eight therapeutic domains (four surgical, four non-surgical), yielding 688 individual decisions. Agreement was summarized descriptively and with Cohen’s κ. **Results:** GPT-5.5 produced complete recommendations for all vignettes. Overall concordance was 90.8% (625/688). Agreement was highest for endocrine therapy, radiotherapy, and targeted therapy (100% each) and for axillary surgery (93.0%, κ  =  0.86), and lowest for breast reconstruction (80.2%), oncoplastic surgery (82.6%) and neoadjuvant chemotherapy (83.7%). Surgical concordance was 85.8% (295/344). Discordances occurred predominantly in individualized surgical decisions; none were observed for endocrine therapy, radiotherapy, or targeted therapy. **Conclusions:** GPT-5.5 showed high overall concordance with real MDT decisions, particularly for guideline-driven systemic therapy, but was less reliable for individualized surgical planning. These findings should be regarded as exploratory and cannot be assumed to be generalizable across institutions or clinical settings. LLMs should complement, not replace, multidisciplinary expertise; prospective multicenter validation is warranted.

## 1. Introduction

Breast cancer remains the most frequently diagnosed malignancy and the leading cause of cancer-related morbidity among women worldwide. Despite substantial advances in screening, molecular characterization, and systemic therapies, optimal management continues to rely on complex, individualized treatment decisions integrating clinical, radiological, pathological, and patient-related factors. International guidelines issued by the European Society for Medical Oncology (ESMO), the National Comprehensive Cancer Network (NCCN), and the St. Gallen International Consensus provide evidence-based recommendations; however, these cannot replace multidisciplinary clinical judgment in situations where multiple therapeutic options are appropriate [1,2,3].

Multidisciplinary team (MDT) meetings represent the current standard of care for breast cancer management. By combining the expertise of breast surgeons, medical oncologists, radiation oncologists, radiologists, pathologists, plastic surgeons, geneticists, and breast nurses, MDTs ensure that treatment recommendations are individualized according to tumor biology, disease stage, technical feasibility, patient comorbidities, and personal preferences. Numerous studies have demonstrated that multidisciplinary decision-making improves adherence to clinical guidelines, optimizes treatment sequencing, and contributes to better oncological outcomes [4]. Nevertheless, MDT discussions are time-consuming, require significant institutional resources, and may be challenged by increasing patient volumes and the growing complexity of modern breast cancer care.

Artificial intelligence (AI) has emerged as one of the most rapidly evolving technologies in medicine. Within breast oncology, AI applications have been successfully developed for mammography interpretation, digital pathology, risk prediction, and workflow optimization [5]. More recently, large language models (LLMs) have introduced a different paradigm of AI by generating context-dependent natural language responses capable of summarizing clinical information, explaining medical concepts, and proposing diagnostic or therapeutic strategies. Among these models, GPT-5.5 represents one of the latest generations of conversational AI with enhanced reasoning capabilities and improved integration of medical knowledge.

Unlike conventional clinical decision-support systems, which operate using predefined algorithms or structured prediction models, LLMs synthesize large amounts of textual information and generate recommendations based on probabilistic language modelling. Their ability to rapidly analyze complex clinical scenarios has generated considerable interest regarding their potential role in supporting oncological decision-making [6]. However, fluent and well-structured responses should not be interpreted as evidence of clinical accuracy. LLMs remain susceptible to hallucinations, incomplete reasoning, inconsistent recommendations and inadequate incorporation of contextual factors that frequently influence real-world treatment decisions.

Several recent studies have evaluated the performance of ChatGPT and other LLMs in breast cancer management. Most have reported moderate-to-high agreement with established guidelines or multidisciplinary recommendations, particularly for standardized treatment scenarios. However, the available evidence remains heterogeneous. Many investigations have relied on simulated clinical vignettes rather than on real patients, included relatively small datasets, evaluated earlier model generations, or assessed isolated therapeutic decisions instead of the complete multidisciplinary management pathway [7,8]. Moreover, agreement with guideline-based recommendations does not necessarily reflect agreement with individualized MDT decisions, which often incorporate patient-specific anatomical, surgical, logistical, and psychosocial considerations that extend beyond published recommendations.

Surgical decision-making represents a particularly challenging domain for AI-assisted recommendations. The choice between breast-conserving surgery and mastectomy, the indication for oncoplastic techniques, axillary management, immediate reconstruction, and treatment sequencing frequently depend on nuanced clinical judgment, imaging interpretation, surgeon experience, institutional expertise, and patient preference. These multidimensional factors are difficult to fully capture within structured textual inputs and may limit the reliability of LLM-generated recommendations despite otherwise accurate interpretation of tumor biology [9].

Consequently, there is a growing need for studies evaluating LLM performance using real-world clinical cases managed according to contemporary multidisciplinary practice. Such investigations are essential to determine not only whether AI-generated recommendations are guideline-concordant, but also whether they reflect the individualized decision-making process that characterizes modern breast cancer care [10,11].

The present study aimed to evaluate the agreement between therapeutic recommendations generated by GPT-5.5 and the reference multidisciplinary management of patients with invasive breast cancer using standardized preoperative clinical information. Agreement was assessed across eight clinically relevant decision domains encompassing both surgical and non-surgical treatment components. By identifying areas of concordance and discordance, this study seeks to provide an exploratory assessment of the potential role and current limitations of LLMs as clinical decision-support tools in breast cancer management, while emphasizing that these technologies are intended to complement rather than replace multidisciplinary expertise.

## 2. Materials and Methods

### 2.1. Study Design and Reporting

This retrospective, single-center observational study evaluated the agreement between therapeutic recommendations generated by GPT-5.5 and the real-world multidisciplinary management of patients with invasive breast cancer. The study was designed as an exploratory proof-of-concept investigation assessing the feasibility of a large language model (LLM) as a clinical decision-support tool in breast cancer management using standardized preoperative clinical information.

The overall study workflow is presented in Figure 1.

### 2.2. Study Population

Patients undergoing surgery for invasive breast cancer between February 2025 and July 2026 at a tertiary breast surgery center in Cluj-Napoca, Romania, were retrospectively screened.

Eligibility criteria included: histologically confirmed invasive breast carcinoma; complete preoperative clinical documentation; available breast imaging; core needle biopsy with complete immunohistochemical assessment; and operative and pathological reports enabling reconstruction of the multidisciplinary therapeutic decision.

Patients were excluded if they presented with pure ductal carcinoma in situ; incomplete clinical documentation; unavailable operative records; or insufficient information to establish the multidisciplinary reference strategy.

Ninety-one consecutive cases were initially reviewed. After applying the predefined eligibility criteria, 86 patients were included in the final analysis. 

### 2.3. Development of Standardised Clinical Vignettes

For each patient, a standardized clinical vignette was created exclusively using information available before therapeutic decision-making (Table 1). The vignette fields were those of the standardized prompt (Supplementary Material, Section S1; Table 1): case number; patient age; tumor location; clinical stage at diagnosis (TNM staging, when documented); most recent preoperative tumor size; nodal status after completing neoadjuvant treatment, when documented; histological subtype; histological grade from core biopsy, when available; estrogen and progesterone receptor status; HER2 status; Ki-67 index; multifocal or multicentric disease known preoperatively; tumor-to-breast-volume ratio, when available; BRCA1/2 status, when available; neoadjuvant treatment received; and history of breast surgery. Preoperative imaging was used to document the corresponding fields (tumor size, multifocal or multicentric disease, nodal status, tumor-to-breast-volume ratio); imaging reports by modality were not transmitted as separate variables. Menopausal status (Table 2) was not part of the vignettes.

To reproduce the clinical information available during multidisciplinary discussion, all postoperative findings were intentionally withheld from the model. These included pathological tumor size, pathological nodal stage, residual disease, pathological response after neoadjuvant therapy, postoperative HER2 reclassification, and all other information unavailable at the time of therapeutic planning. 

### 2.4. GPT-5.5 Evaluation Protocol

ChatGPT-5.5 (OpenAI, San Francisco, CA, USA) was evaluated using a standardized frozen prompting framework designed for multidisciplinary breast cancer decision support (Supplementary Material, Section S1). The prompt was based on the predefined clinical variables described in Table 1 and presented each case as a standardized hypothetical clinical scenario. GPT-5.5 was asked to provide one treatment recommendation across eight predefined clinical domains. Before the formal study, multiple pilot interactions were performed to optimize prompt clarity and response consistency. These preliminary sessions were used exclusively for prompt refinement and were not included in the final analysis.

The evaluations were performed on 10–11 July 2026 using GPT-5.5 in Medium reasoning mode. Web Search was retained, as this study aimed to evaluate the model under a clinically realistic configuration in which contemporary and potentially updated medical information could be retrieved dynamically.

To maximize reproducibility and minimize response variability, every case was evaluated using the identical frozen prompt, under identical model settings, in a new conversation (without access to previous patient cases). However, because Web Search was enabled, the external information retrieved by the model might change over time as online sources are updated, modified, or removed. Even though the prompt, model configuration and input clinical information were standardized, exact replication of the generated responses cannot be guaranteed later. This represents a limitation of evaluating an LLM with dynamic web-access capabilities and was taken into consideration when interpreting the reproducibility of the present findings.

The model was instructed to assume the role of an experienced breast surgical oncologist participating in a multidisciplinary tumor board. Therapeutic recommendations were requested according to contemporary ESMO, NCCN, and St. Gallen International Consensus recommendations while considering the individual clinical scenario.

To reduce model-related variability, no follow-up prompts, iterative refinements, or clarification requests were permitted after the initial response. GPT-5.5 generated a single recommendation for each clinical vignette, thereby simulating a first-pass multidisciplinary decision-support scenario.

The model was only provided with clinical data, imaging, and biopsy results that were documented and available prior to the surgical decision (the investigator-side “known before the decision” rule, Supplementary Material, Section S2).

### 2.5. Multidisciplinary Reference Standard

GPT-5.5 recommendations were compared with a multidisciplinary reference standard reflecting real-world clinical practice rather than guideline interpretation alone. The reference management strategy represented the therapeutic decisions reached through routine multidisciplinary evaluation and therefore incorporated clinical, radiological, pathological, surgical, technical, and patient-related factors that may not be fully represented within guideline algorithms.

For the surgical domains, the reference standard corresponded to the procedures performed and documented in operative reports, including breast surgery, use of oncoplastic techniques, axillary surgery and breast reconstruction.

For systemic therapy and radiotherapy, the reference standard was established according to contemporary international guidelines and confirmed by the coordinating breast surgeon based on the complete multidisciplinary clinical context.

The multidisciplinary reference standard should not be regarded as an objective or universally applicable gold standard. MDT decisions may be based on institution-specific treatment pathways, available resources, local expertise, surgeon experience and individual clinician preferences (for instance, in areas where multiple clinically acceptable management strategies exist). Patient preferences and shared decision-making could also contribute to variation in selecting the appropriate treatment. The discordance between GPT-5.5 and the MDT reference decision does not necessarily imply that the AI-generated recommendation was clinically inappropriate, and concordance should not be interpreted as definitive evidence of clinical correctness.

GPT-5.5 generated all recommendations without prior knowledge of the reference management strategy.

### 2.6. Outcome Measures

Agreement between GPT-5.5 and the multidisciplinary reference standard was evaluated across eight predefined therapeutic domains. Surgical domains included breast surgery type (breast conserving or mastectomy), use of an oncoplastic procedure, axillary surgery (sentinel lymph node biopsy, targeted axillary dissection, or axillary clearance), and breast reconstruction (4 items). Non-surgical domains included endocrine therapy, radiotherapy, neoadjuvant chemotherapy, and targeted therapy (anti-HER2) together with immunotherapy (4 items).

Each domain was classified as either concordant (1 point) or discordant (0 points).

Three summary scores were subsequently calculated: Surgical Concordance Score (0–4), Non-surgical Concordance Score (0–4), and Overall Concordance Score (0–8).

Because this exploratory pilot study focused on overall agreement, all discordances were initially treated as binary outcomes. Nevertheless, discordant recommendations were further reviewed qualitatively according to their potential clinical relevance.

### 2.7. Statistical Analysis

Owing to the exploratory design and rather limited sample size, statistical analyses were primarily descriptive. Categorical variables are presented as frequencies and percentages. Agreement was calculated for each individual patient, for each therapeutic domain, separately for surgical and non-surgical management, and for the overall therapeutic strategy. Inter-observer agreement between GPT-5.5 and the multidisciplinary reference standard was additionally quantified using the Cohen’s κ coefficient for each therapeutic domain and interpreted according to the criteria of Landis and Koch.

Continuous variables are reported as median and interquartile range (IQR) or mean ± standard deviation, as appropriate. Formal hypothesis testing was not performed because the study was not powered for inferential analyses.

Statistical analyses were conducted using Python version 3.12.3.

### 2.8. Ethical Considerations and AI Use

The present study was approved by the ethics committee of Cluj County Emergency Clinical Hospital, Cluj-Napoca, Romania (Approval No. 32024/2 September 2026).

To ensure patient privacy and data minimization, no original medical records, reports, or identifiable patient information were entered into GPT-5.5. The investigators converted each real-world case into a standardized, de-identified clinical vignette (a clinical scenario derived from the corresponding patient case). Only medical information mandatory for the predefined research objectives was retained, and all direct identifiers and non-essential potentially identifying information were excluded. The AI system therefore received only de-identified clinical information required to reproduce the relevant preoperative decision-making context and had no access to original patient records or institutional databases. The generated recommendations were used only for research comparison and did not influence clinical management or patient care.

GPT-5.5 (OpenAI, San Francisco, CA, USA) was used as the investigational tool evaluated in this study. The model generated therapeutic recommendations based on standardized, de-identified preoperative clinical vignettes using a frozen prompt, Medium reasoning mode, and Web Search enabled. The AI-generated recommendations were subsequently compared with the corresponding multidisciplinary team MDT decisions across predefined surgical and non-surgical treatment domains. The model was used exclusively for research purposes and was not involved in patient care, clinical decision-making, data collection or the analysis of patient outcomes.

In addition, ChatGPT (OpenAI) was used during manuscript preparation for minor editorial assistance, including language polishing, grammar correction, sentence rephrasing, improvement of clarity and readability and formatting of selected sections.

All AI-assisted outputs were critically reviewed, verified, and revised by the authors. The authors take full responsibility for the accuracy, integrity, and final content of the manuscript. 

## 3. Results

### 3.1. Patient Characteristics

A total of 91 consecutive breast cancer cases were retrospectively screened for eligibility. Following the application of the predefined inclusion and exclusion criteria, 86 patients with invasive breast carcinoma were included in the final analysis (Figure 1).

All included cases contained sufficient preoperative clinical, radiological, pathological, and operative documentation to allow reconstruction of the multidisciplinary therapeutic context. Standardized clinical vignettes were created for each patient exclusively using preoperative information available at the time of treatment planning.

The clinicopathological characteristics of the study population are summarized in Table 2.

### 3.2. Concordance Between GPT-5.5 and the Multidisciplinary Reference Standard

A total of 688 therapeutic decisions (86 patients × 8 predefined domains) were evaluated. GPT-5.5 generated recommendations for all clinical vignettes without technical failure or incomplete responses. Overall agreement with the multidisciplinary reference standard varied across individual patients and therapeutic domains.

### 3.3. Surgical Concordance

Agreement within the four surgical domains (breast surgery, oncoplastic surgery, axillary surgery, and breast reconstruction) showed variability between cases. The highest agreement was observed for standard surgical procedures with clearly defined indications, whereas recommendations requiring individualized surgical judgment demonstrated greater variability. Domain-level surgical concordance is summarized in Table 3.

### 3.4. Concordance for Systemic Treatment Recommendations

Agreement for endocrine therapy, radiotherapy, and targeted therapy/immunotherapy was generally higher than that observed for surgical management. Recommendations based primarily on tumor biology and current guideline-directed systemic treatment algorithms demonstrated the greatest consistency with the multidisciplinary reference standard. Detailed concordance for each therapeutic domain is presented in Table 3.

### 3.5. Overall Concordance

Comparison of Cohen’s κ coefficients demonstrated differences in the level of agreement among therapeutic domains (Table 3).

Across all evaluated therapeutic domains, GPT-5.5 generated 625 concordant recommendations out of 688 individual treatment decisions, corresponding to an overall concordance rate of 90.8%. Overall agreement varied according to the complexity of the therapeutic domain. The highest concordance was observed for endocrine therapy, radiotherapy, and targeted therapy, for which GPT-5.5 achieved complete agreement with MDT recommendations (100% in each domain). Among surgical decisions, the highest concordance was obtained for axillary management (93.0%), followed by breast surgery (87.2%). Lower agreement was observed for neoadjuvant chemotherapy (83.7%), oncoplastic surgery (82.6%), and immediate breast reconstruction (80.2%).

When only surgical decision-making was considered, GPT-5.5 correctly reproduced 295 of 344 MDT recommendations, yielding an overall surgical concordance of 85.8%. This finding suggests that the model performs particularly well in standardized surgical decisions, such as axillary management, while greater variability occurs in procedures requiring individualized reconstructive planning and surgeon-specific expertise.

Overall, these results indicate that, within this single-center cohort, GPT-5.5 frequently generated recommendations that were concordant with MDT treatment decisions across a broad spectrum of breast cancer treatment domains. The highest reliability was observed for evidence-based systemic therapies and the greatest variability in complex surgical scenarios where patient anatomy, cosmetic considerations, and surgeon experience play a central role.

Notably, despite evaluating eight therapeutic domains for each patient, GPT-5.5 achieved an overall concordance exceeding 90%, highlighting its ability to integrate clinical, imaging-based and pathological information into recommendations that closely resemble multidisciplinary expert decision-making. However, the findings should be interpreted cautiously considering the limited sample size and single-center design. The remaining discordances were predominantly observed in individualized surgical decisions rather than in guideline-driven systemic treatment recommendations.

### 3.6. Analysis of Discordant Recommendations

Discordant recommendations were identified in both surgical and non-surgical treatment domains. Most discrepancies occurred in clinical scenarios requiring individualized multidisciplinary assessment rather than direct application of guideline recommendations. The most frequent areas of disagreement involved selection of breast-conserving surgery versus mastectomy, indication for oncoplastic techniques, axillary surgical management in complex clinical scenarios, and immediate breast reconstruction in selected patients.

Among the 49 discordant surgical recommendations, most discrepancies involved indications for immediate breast reconstruction (17/49, 34.7%), oncoplastic surgery (15/49, 30.6%), and breast surgery type (11/49, 22.4%), whereas only six discrepancies (6/49, 12.2%) were observed for axillary surgery. Among non-surgical domains, discordances were confined to neoadjuvant chemotherapy (14 cases); no discordant recommendations were identified for endocrine therapy, radiotherapy, or targeted therapy (Table 4).

## 4. Discussion

### 4.1. Interpretation of the Main Findings and Overview of the Existing Literature

The rapid evolution of large language models (LLMs) has generated considerable interest regarding their potential role in supporting clinical decision-making across multiple medical specialties. Breast cancer management represents a particularly demanding clinical setting because treatment decisions require integration of tumor biology, imaging findings, surgical feasibility, multidisciplinary expertise, and patient preferences [12]. The present study evaluated the agreement between GPT-5.5-generated therapeutic recommendations and real-world multidisciplinary management using standardized preoperative clinical information.

The principal finding of this study is that GPT-5.5 demonstrated variable agreement across therapeutic domains. Recommendations based primarily on standardized biological characteristics and established guideline-directed therapies showed higher concordance with the multidisciplinary reference standard, whereas recommendations requiring individualized surgical planning exhibited greater variability. These findings suggest that current LLMs may provide valuable support for structured, evidence-based treatment components but remain less reliable when complex surgical judgment and contextual clinical reasoning are required. Our findings are consistent with the emerging literature evaluating LLMs in oncology. Several recent studies have reported moderate-to-high agreement between ChatGPT-generated recommendations and guideline-based management strategies. However, most published investigations [13,14,15] relied on simulated clinical vignettes or simplified patient scenarios rather than real-world multidisciplinary cases. Consequently, they primarily assessed guideline adherence rather than the more nuanced clinical reasoning that characterizes multidisciplinary decision-making. By evaluating actual patients discussed in routine clinical practice, our study provides a complementary perspective that better reflects everyday breast cancer management.

An important observation is the difference in performance between surgical and non-surgical treatment domains. Systemic therapies such as endocrine treatment, targeted therapy, immunotherapy, and radiotherapy are generally determined by well-defined clinicopathological variables and are supported by robust international guidelines. These decisions can therefore be more readily reproduced by an LLM trained on large biomedical corpora [16,17,18].

In contrast, surgical planning frequently depends on factors that are difficult to fully encode in textual clinical summaries [19,20,21]. The choice between breast-conserving surgery and mastectomy, the indication for oncoplastic techniques, axillary management, or immediate reconstruction often requires detailed interpretation of imaging, assessment of the breast-to-tumor volume ratio, technical feasibility, cosmetic considerations, institutional expertise, and patient expectations [22]. These multidimensional aspects explain why lower agreement was observed in individualized surgical decisions despite accurate interpretation of biological tumor characteristics.

Our results therefore reinforce the concept that LLMs should currently be considered clinical decision-support tools rather than autonomous decision-makers. GPT-5.5 demonstrated the ability to organize clinical information, identify relevant therapeutic options, and generate recommendations that were frequently consistent with contemporary guidelines. Nevertheless, guideline concordance alone does not guarantee clinical appropriateness for individual patients. Real-world multidisciplinary decisions incorporate contextual information that frequently remains unavailable to language models and may legitimately lead to management strategies that differ from guideline-derived recommendations while remaining clinically appropriate [23,24,25].

### 4.2. Strengths and Limitations of the Current Study

The present study has several important strengths. 

Unlike many previous investigations, it evaluated consecutive real-world breast cancer cases rather than hypothetical scenarios [26,27]. Recommendations were compared with actual multidisciplinary management rather than isolated guideline interpretations, allowing assessment of clinically meaningful agreement. 

Moreover, surgical and non-surgical treatment components were analyzed separately, providing a more detailed understanding of the domains in which LLMs currently perform well and those that continue to require expert multidisciplinary judgment.

Another noteworthy aspect of the present study is the methodological approach. To improve reproducibility, all clinical cases were evaluated using identical standardized preoperative vignettes, a frozen prompt, identical model settings, and independent conversations for each patient. This design minimized prompt-related variability and reduced one of the major methodological limitations affecting previous LLM evaluation studies. Furthermore, only information available before therapeutic decision-making was provided to the model, preventing retrospective bias introduced by postoperative findings.

Despite these strengths, several limitations should be acknowledged. 

First, this was a retrospective, single-center exploratory study in which we included a relatively small cohort of 86 patients. The limited sample size reduces the precision of the estimated concordance rates. Therefore, the observed level of agreement should not be interpreted as representative of MDT decision-making more broadly. Larger prospective multicenter studies involving more diverse patient populations are needed to assess the reproducibility and generalizability of these findings. 

Second, the reference standard reflected real-world multidisciplinary management rather than an external adjudication panel; although this approach increases clinical relevance, some treatment decisions inevitably incorporate institutional preferences and surgeon experience. Treatment decisions can depend on institutional protocols, availability of specific treatments, local surgical expertise and patient-specific considerations that are not necessarily captured by international guidelines. This is particularly relevant for surgical domains like oncoplastic surgery and breast reconstruction, where several approaches could be clinically reasonable. Thus, agreement with the MDT decision should be regarded as concordance with local real-world practice rather than as proof of objective clinical correctness. On the other hand, a discordant LLM recommendation should not automatically be regarded as clinically erroneous when alternative evidence-based strategies may be appropriate. 

Third, GPT-5.5 was evaluated using one standardized prompt and one model configuration. Although a frozen prompt was used, together with similar settings, independent conversations and a predefined evaluation period improved methodological consistency, the activation of Web Search introduces an important limitation with respect to AI reproducibility. The external information available to the model is dynamic and might change as websites and online medical resources are updated, modified, or even removed. Hence, an identical clinical vignette submitted at a later date might not produce the same response, even when the same prompt and model configuration are used. This limitation should be considered when interpreting the reported concordance rates and represents an important methodological challenge for the reproducibility of LLM-based clinical research. 

Fourth, the neoadjuvant chemotherapy domain should be given particular caution. The vignettes indicated whether a neoadjuvant treatment had already been administered, because this information is necessary for reasoning about surgery after neoadjuvant therapy, and the reference for this domain was whether neoadjuvant chemotherapy had been administered. The model’s answer to the question “Would you have proposed neoadjuvant chemotherapy?” may therefore have been anchored by prior-treatment information, leading to a potential information-leakage bias.

Finally, the study assessed agreement with therapeutic decisions rather than patient outcomes and therefore cannot determine whether AI-supported recommendations would improve clinical effectiveness or safety.

### 4.3. Future Directions

Future research should focus on prospective multicenter validation involving larger patient cohorts and multiple institutions. Comparative evaluation of different LLMs, assessment of inter-model reproducibility, incorporation of information including breast imaging, and evaluation of clinical workflow integration will be essential before routine implementation can be considered [28,29,30]. 

Moreover, future studies should investigate whether AI-assisted multidisciplinary meetings improve efficiency, reduce decision variability, or facilitate adherence to evidence-based recommendations without compromising patient safety.

The next step for clinical integration of LLMs could be represented by explainable artificial intelligence (XAI); by improving transparency regarding the factors and evidence underlying AI-generated recommendations, XAI might optimize clinician evaluation, informed decision-making and accountability [31]. This can be particularly relevant in the field of breast cancer, in which treatment decisions usually involve multiple clinical and patient-related factors. Future LLM-based clinical decision-support systems should be assessed not only for concordance with MDT decisions, but also for explainability, transparency and safe human oversight.

Overall, our findings support a pragmatic view of artificial intelligence in breast oncology. GPT-5.5 appears capable of assisting clinicians by synthesizing clinical information and generating structured treatment recommendations. However, current LLMs do not replace multidisciplinary expertise [32]. Instead, their greatest value may lie in supporting—not substituting—the complex clinical reasoning that remains fundamental to personalized breast cancer care. Moreover, as disease complexity increases, LLMs show a diminished capacity to tailor recommendations to individual patients, failing to incorporate real-world factors (such as comorbidities, financial burden, or treatment tolerance) that clinicians routinely balance against guideline-based protocols [33,34].

Artificial intelligence should be viewed not as a replacement for multidisciplinary expertise, but as a promising technology capable of augmenting evidence-based, patient-centered breast cancer care while preserving clinical responsibility and human judgment [35,36,37].

## 5. Conclusions

In this retrospective, single-center exploratory study of 86 patients with invasive breast cancer, GPT-5.5 showed an increased level of agreement with real-world MDT treatment decisions (when standardized preoperative clinical information was provided). Agreement was generally higher for treatment components driven by well-established clinicopathological criteria and international guideline recommendations than for individualized surgical decisions requiring multidisciplinary clinical judgment.

These findings suggest that large language models have the potential to function as clinical decision-support tools by assisting clinicians in organizing patient information, summarizing evidence-based therapeutic options, and facilitating multidisciplinary discussions. However, current LLMs should not be regarded as autonomous decision-makers, particularly in complex surgical scenarios where anatomical considerations, technical feasibility, institutional expertise, and patient preferences play a decisive role.

The present study supports the concept that artificial intelligence should complement—not replace—multidisciplinary breast cancer teams. Careful human supervision remains essential to ensure the safety, appropriateness, and personalization of treatment recommendations.

Future prospective multicenter studies involving larger patient cohorts, external validation and AI systems integrating clinical, imaging, and pathological data are needed to better define the role of LLMs within routine breast cancer care. Such investigations should evaluate not only agreement with multidisciplinary decisions but also their impact on workflow efficiency, decision consistency, patient outcomes, and healthcare resource utilization.

## Figures and Tables

**Figure 1:**
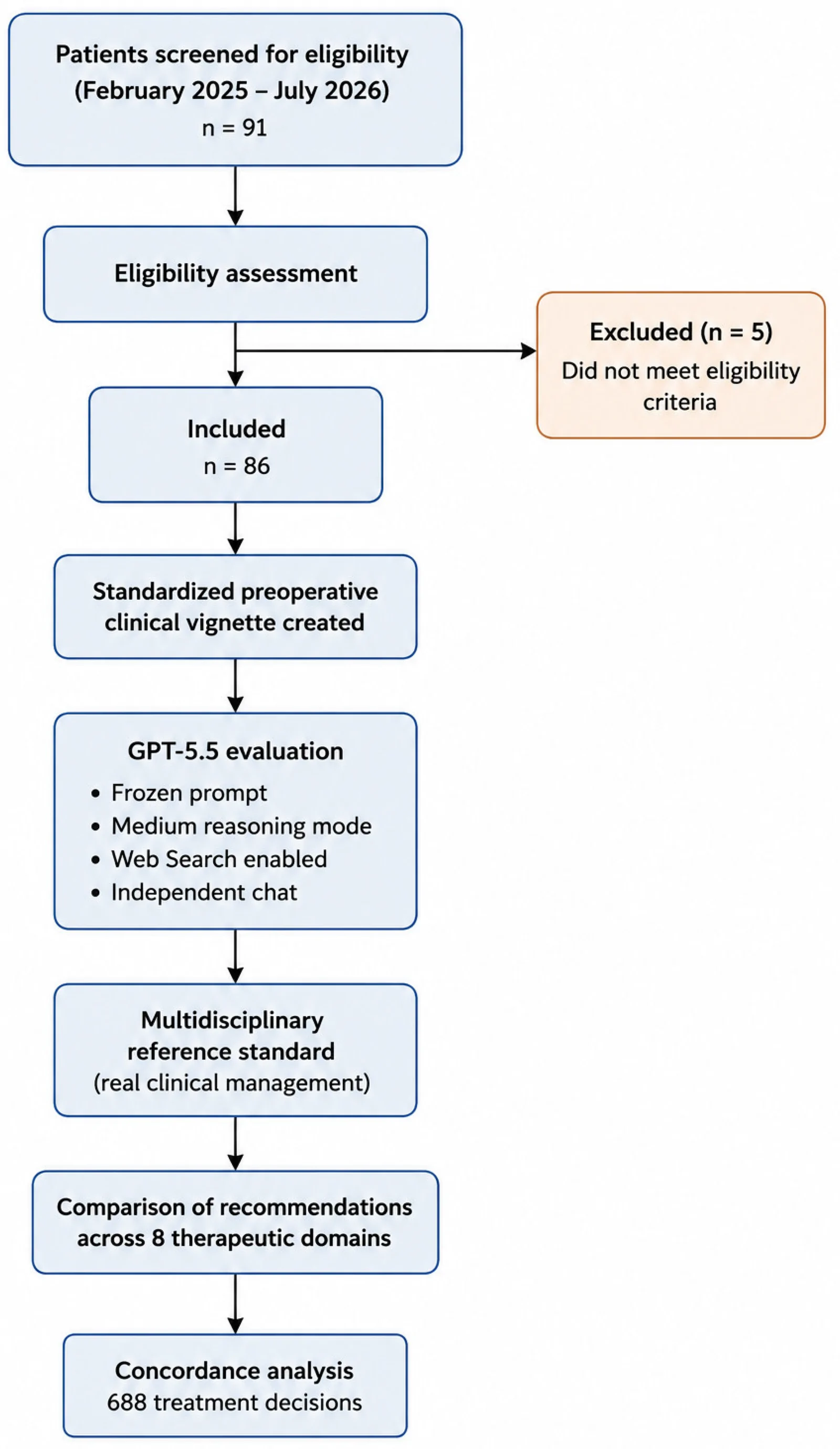
Study workflow: A total of 91 consecutive patients were screened for eligibility. After exclusion of 5 cases that did not meet the predefined eligibility criteria, 86 patients with invasive breast cancer were included. Standardized preoperative clinical vignettes were independently assessed by GPT-5.5 using a frozen prompt, Medium reasoning mode, and Web Search enabled. AI-generated recommendations were then compared with the MDT recommendations across 8 predefined therapeutic domains, yielding 688 treatment decisions for concordance analysis.

**Table 1:** Clinicopathological information used for the development of standardized clinical vignettes.

Section	Variables Included
Patient	Age; history of breast surgery; BRCA1/2 status (when available)
Clinical	Tumor location; clinical stage at diagnosis (cT, cN, cM, when documented)
Preoperative tumor assessment (based on imaging)	Most recent preoperative tumor size; multifocal or multicentric disease known before surgery; tumor-to-breast-volume ratio (when available); nodal status after neoadjuvant treatment (when documented)
Pathology	Histological type; grade (when available); ER; PR; HER2; Ki-67
Previous treatment	Neoadjuvant treatment
GPT-5.5 output	Breast surgery; surgical technique; oncoplastic surgery; breast reconstruction; axillary surgery; endocrine therapy; radiotherapy; targeted therapy/immunotherapy; neoadjuvant chemotherapy proposal; consistency with guidelines

**Table 2:** Baseline clinicopathological characteristics of the 86 patients included in the study.

Characteristic	Value
Age, years	48.6 ± 12.4 (range 19–78)
Menopausal status	
Premenopausal	39 (45.34%)
Postmenopausal	47 (54.65%)
Clinical T stage	
cT1	22 (25.58%)
cT2	42 (48.83%)
cT3	16 (18.60%)
cT4	6 (6.97%)
Clinical nodal stage	
cN0	49 (56.97%)
cN1	28 (32.55%)
cN2–3	9 (10.46%)
Histological subtype	
Invasive carcinoma of no special type (NST)	57 (66.27%)
Invasive lobular carcinoma	18 (20.93%)
Other	11 (12.79%)
Histological grade	
Grade 1	15 (17.44%)
Grade 2	47 (54.65%)
Grade 3	24 (27.90%)
Receptor and HER2 status	
ER positive	51 (59.30%)
PR positive	49 (56.97%)
HER2 positive	38 (44.18%)
Ki-67 ≥ 20%	56 (65.11%)
Intrinsic subtype	
Luminal A-like	14 (16.27%)
Luminal B/HER2 negative	12 (13.95%)
Luminal B/HER2 positive	25 (29.06%)
HER2 enriched	13 (15.11%)
Triple negative	22 (25.58%)
Multifocal disease	6 (6.97%)
Multicentric disease	7 (8.13%)
Neoadjuvant systemic therapy	60 (69.76%)

Continuous variables are presented as mean ± standard deviation; categorical variables as number (percentage). Intrinsic molecular subtypes were classified according to immunohistochemical surrogate definitions.

**Table 3:** Agreement between GPT-5.5 and the multidisciplinary team across the eight therapeutic domains.

Therapeutic Domain	Concordant, n	Discordant, n	Concordance rate, %	Cohen’s κ
Breast surgery	75	11	87.2	0.74
Oncoplastic surgery	71	15	82.6	0.65
Axillary surgery	80	6	93.0	0.86
Breast reconstruction	69	17	80.2	0.60
Radiotherapy	86	0	100.0	1.00
Endocrine therapy	86	0	100.0	1.00
Neoadjuvant chemotherapy	72	14	83.7	0.67
Targeted therapy/immunotherapy	86	0	100.0	1.00

Cohen’s κ interpreted according to Landis and Koch. Overall concordance: 625/688 decisions (90.8%); surgical concordance: 295/344 decisions (85.8%).

**Table 4:** Distribution of discordant recommendations across therapeutic domains.

Therapeutic Domain	Discordant Recommendations, n (%)
Breast reconstruction	17 (19.76%)
Oncoplastic surgery	15 (17.44%)
Neoadjuvant chemotherapy	14 (16.27%)
Breast surgery	11 (12.79%)
Axillary surgery	6 (6.97%)
Endocrine therapy	0 (0%)
Radiotherapy	0 (0%)
Targeted therapy	0 (0%)

Percentages are calculated relative to the 86 patients included in the study.

## Data Availability

The main data supporting the findings of this study are available within the article and its Supplementary Materials.

## Supplementary Materials

The following supporting information can be downloaded at: https://www.mdpi.com/article/10.3390/jcm15197533/s1. Standardized Prompting Framework for GPT-5.5 Evaluation (Section S1) and “Known before the decision” rule and concordance scoring grid (Section S2). English translation and adaptation of the standardized prompt used for the final AI-based assessments, originally developed and included as an appendix to the undergraduate thesis entitled “Intelligence Artificielle dans la prise en charge du cancer du sein: Analyse comparative des décisions thérapeutiques et perception médico-sociale” authored by Noé Yoshi François Poupel, defended in 2026 and conducted under the scientific supervision of Alexandra Caziuc, at the “Iuliu Hațieganu University of Medicine and Pharmacy”, Cluj-Napoca, Romania. The present version has been translated and adapted for the purposes of the current study.
