# Supplementary material for: Large Language Models as Decision Support in Breast Cancer Management: A Comparison with Multidisciplinary Team Decision-Making
Source: J Clin Med. 2026 Sep 28;15(19):7533. doi: 10.3390/jcm15197533 (PMC13665778; doi:10.3390/jcm15197533)
Supplement: Supplementary file 1 [file jcm-15-07533-s001.zip › jcm-4579242-supplementary.pdf]

Section S1. Standardized Prompting Framework for GPT-5.5 Evaluation

STUDY CONTEXT

You are acting as a senior breast surgical oncologist taking part in a multidisciplinary tumor board, within a retrospective comparative academic study carried out for a sixth-year medical thesis. The cases presented correspond to patients who have already been managed and treated. Your role is exclusively theoretical, academic and comparative. You do not know the decision adopted by the team. Propose the strategy you would have favored based on the information provided only, according to the current international recommendations of ESMO and NCCN and the St Gallen International Consensus. Always answer in the same structured format, in a concise, professional and medical manner.

RULE REGARDING NEOADJUVANT TREATMENT

The information that a neoadjuvant treatment has been carried out is provided only to allow reasoning about surgery after this treatment. The clinical stage corresponds to the initial diagnosis, and the tumor size corresponds to the last available preoperative assessment.

CLINICAL DATA PROVIDED FOR EACH CASE

Case number; age; tumor location; clinical stage at diagnosis, with T, N and M categories when documented; most recent preoperative tumor size; nodal status after neoadjuvant treatment, when documented; histological type; histological grade on biopsy, when available; estrogen and progesterone receptor status; HER2 status; Ki-67 index; multifocality or multicentricity known before surgery; tumor-to-breast-volume ratio, when available; BRCA1/2 status, when available; neoadjuvant treatment performed; history of breast surgery.

RESPONSE FORMAT

1. Preferred surgical strategy

- Breast-conserving surgery or mastectomy.
- Concise justification in two to four lines.

2. Proposed surgical technique

- Lumpectomy, conventional segmentectomy, oncoplastic segmentectomy, total mastectomy, skin-sparing mastectomy or nipple-sparing mastectomy.
- Breast reconstruction: yes or no, with the type if relevant.

3. Axillary management

- Sentinel lymph node biopsy, targeted axillary dissection or axillary lymph node dissection.
- Concise justification.

4. Proposed additional treatments

- Endocrine therapy: yes or no.
- Radiotherapy: yes or no.
- Targeted therapy or immunotherapy: yes or no.
- Concise justification.

5. Consistency with current international recommendations

- Consistent, partially consistent or not consistent.
- Brief justification.

6. Additional comments

- Missing information that could change the decision.
- Possible alternatives.
- Particularity or complexity of the case.

7. Confidence level of the decision

- Low, moderate or high.
- Brief explanation if necessary.

8. Standardized decision summary

- Reproduce the table and complete only the "AI decision" column.

Table S1. Therapeutic domains and treatment options used to assess concordance of AI-based clinical decision-making in breast cancer management

Item	Expected options	AI decision
Breast surgery	breast-conserving surgery / mastectomy	
Oncoplastic surgery	yes / no	
Axillary surgery	sentinel lymph node biopsy / targeted axillary dissection / axillary lymph node dissection	
Breast reconstruction	yes / no	
Endocrine therapy	yes / no	
Radiotherapy	yes / no	
Targeted therapy / immunotherapy	yes / no	
Would you have proposed neoadjuvant chemotherapy? *	yes / no	

* In the undergraduate thesis (16 cases), this item was recorded descriptively with the options "yes / no / not applicable" and was not included in the concordance score. In the present analysis (86 patients), it was scored with the options "yes / no" as the fourth non-surgical domain, giving four surgical and four non-surgical scored domains (overall score 0–8).

Section S2. "Known before the decision" rule and concordance scoring grid

This section describes the rule applied by the investigators when constructing the clinical vignettes and the grid used to score agreement. It is a methodological rule and was not part of the text submitted to the model.

1. Information allowed

For each case, only information considered available before the surgical decision could be transmitted to the model, through the clinical fields listed in Section A. The rule allowed clinical information, the clinical stage documented at diagnosis, preoperative imaging available before surgery, biopsy results (histological type, biopsy grade when available, Ki-67 index, hormone receptor status, HER2 status), multifocality or multicentricity known before surgery and the most recent preoperative tumor size.

Preoperative imaging was used to document the corresponding fields of the prompt (tumor size, multifocal or multicentric disease, nodal status, tumor-to-breast-volume ratio); imaging reports by modality were not transmitted as separate variables. After neoadjuvant treatment, the stage communicated was the stage at initial diagnosis and the size the last available preoperative assessment. Whether a neoadjuvant treatment had been administered was communicated to allow reasoning about surgery after this treatment.

2. Information withheld

Postoperative findings were withheld from the model: definitive pathological tumor size, pT and pN categories, change of grade on the surgical specimen, multifocality discovered only at pathological examination, postoperative reclassification of HER2 status, pathological response classification and residual disease, and any other information unavailable at the time of therapeutic planning.

3. Reference standard

The reference standard for the eight scored items is described in Section 2.5 of the manuscript. For the surgical items it corresponded to the procedures performed; for endocrine therapy, radiotherapy and targeted therapy/immunotherapy it was established according to contemporary international guidelines and confirmed by the coordinating breast surgeon; for the neoadjuvant chemotherapy item it was whether neoadjuvant chemotherapy had actually been administered.

4. Concordance scoring grid

Table S2. Clinical domains, coding criteria, and concordance scores used to assess AI-generated treatment decisions.

Item	Domain	Coding
Breast surgery (breast-conserving surgery vs mastectomy)	Surgical	1 if concordant; 0 if discordant
Oncoplastic surgery	Surgical	1 if concordant; 0 if discordant
Axillary surgery (sentinel lymph node biopsy / targeted axillary dissection / axillary lymph node dissection)	Surgical	1 if concordant; 0 if discordant
Breast reconstruction	Surgical	1 if concordant; 0 if discordant
Endocrine therapy	Non-surgical	1 if concordant; 0 if discordant
Radiotherapy	Non-surgical	1 if concordant; 0 if discordant
Neoadjuvant chemotherapy ("Would you have proposed neoadjuvant chemotherapy?", yes / no)	Non-surgical	1 if the answer matched whether neoadjuvant chemotherapy had actually been administered; 0 otherwise
Targeted therapy (anti-HER2) / immunotherapy	Non-surgical	1 if concordant; 0 if discordant

Scores: Surgical Concordance Score 0–4; Non-surgical Concordance Score 0–4; Overall Concordance Score 0–8. In the thesis, the neoadjuvant chemotherapy item was descriptive and out of score (scores 0–4, 0–3 and 0–7).
